# Early post-transplant ventilation and perfusion assessment using phase-resolved functional lung MRI

**DOI:** 10.1007/s11547-026-02274-1

**Published:** 2026-09-18

**Authors:** Marc-Luca Heinze, Fabio Ius, Andreas Voskrebenzev, Till Kaireit, Marius Klein, Arjang Ruhparwar, Jawad Salman, Frank Wacker, Jens Vogel-Claussen, Gesa H. Pöhler

**Affiliations:** 1 Department of Diagnostic and Interventional Radiology, Hannover Medical School, Carl-Neuberg-Str. 1, 30625 Hannover, Germany; 2 Biomedical Research in Endstage and Obstructive Lung Disease Hannover (BREATH), German Center for Lung Research (DZL), Hannover, Germany; 3 Department of Cardiothoracic, Transplant, and Vascular Surgery, Hannover Medical School, Hannover, Germany; 4 Clinic for Radiology, University of Münster and University Hospital of Münster, Münster, Germany

**Keywords:** Lung transplantation, Phase-resolved functional lung (PREFUL) MRI, Pulmonary function testing, Quantified ventilation–perfusion, Regional ventilation-perfusion

## Abstract

**Purpose:**

Post-transplant functional lung surveillance relies on global ventilatory assessment using spirometry, lacking regional ventilation and perfusion measures. We aimed to assess early post-transplant lung function in clinically stable recipients using phase-resolved functional lung (PREFUL) MRI.

**Material and methods:**

In this prospective, single-center study (March 2022–December 2024), clinically stable bilateral lung transplant recipients and 1:1 age- (± 5 years) sex-matched participants (*n* = 100) underwent PREFUL MRI and spirometry. Ventilation and perfusion MRI was voxel-based quantified: Regional ventilation (RVent, [mL/mL]), flow-volume loop ventilation (FVL, [au]) and perfusion (Q, [mL/min/100 mL]). Whole lung defect maps (VDP, QDP [%]), V/Q ratios (%) based on RVent, FVL and Q were generated. Median MRI parameters were compared with controls, spirometry and perioperative data (Bonferroni correction P<.0045 considered significant).

**Results:**

84 participants (42 transplant recipients and 42 healthy participants) were evaluated at median 16 days post-transplantation. In lung transplant recipients RVent, FVL and Q did not differ from controls (all *P *> 0.01). Transplant recipients demonstrated increased RVent VDP (10% vs 3.7%, *P *< 0.001), VDP_combined_ (14% vs 7.1%, *P *< 0.001) and QDP (7.9% vs 2.4%, *P *< 0.001). In transplant recipients FVL, FVL VDP, QDP and V/Q matched defects correlated with spirometry. QDP was associated with prolonged cold ischemic times of first lung allografts (*ρ* = 0.43, *P* = 0.004), V/Q mismatch with cold ischemic times of first and second implanted lungs (*ρ* = 0.48, *P* = 0.001; *ρ* = 0.46, *P *= 0.002).

**Conclusion:**

Free-breathing phase-resolved functional lung MRI-derived parameters revealed increased regional ventilation and perfusion heterogeneity in stable lung transplant recipients two weeks post-transplantation and correlated with spirometry and prolonged cold ischemic times of allografts.

## Introduction

Lung transplantation has become a life-saving therapy for patients with end-stage lung disease, offering substantial improvements in survival and quality of life [1, 2]. However, post-transplant survival remains limited at approximately 6.2 years [3], with 20–30% of deaths occurring beyond the first year [4].

Beyond immunological matching, several perioperative factors have been implicated in immune activation and allograft injury following lung transplantation. Prolonged ischemic time and advanced donor age are independently associated with increased rejection risk, likely through augmentation of innate immune responses and ischemia–reperfusion injury [5–7]. Additional factors, including suboptimal donor quality, preservation solution composition, intraoperative hemodynamic instability, allogeneic blood transfusions and the use of extracorporeal membrane oxygenation, have been linked to primary graft dysfunction and systemic inflammatory activation, which may potentially reduce the threshold for subsequent acute rejection [8, 9].

Early clinical manifestations of rejection are often nonspecific, most commonly including dyspnea, cough or peripheral edema. Pulmonary function testing (PFT) remains the standard tool for monitoring graft function. However, it is effort-dependent, lacks the spatial resolution required to detect focal or regional ventilatory impairment and provides no measure of pulmonary perfusion. These limitations impede differentiation between expected postoperative recovery and early stages of graft rejection, thereby motivating the need for complementary regional, perfusion-sensitive imaging techniques [10]. Although transbronchial biopsy remains the gold standard for diagnosing rejection when spirometry indicates decline, it suffers from inherent sampling bias and may fail to capture regional, spatially heterogeneous rejection patterns, necessitating comprehensive regional functional assessments to enable precision-guided therapeutic approaches [11]. CT serves a supportive role by helping to exclude alternative causes such as infection, edema or airway complications; however, its diagnostic specificity for rejection is low and cumulative radiation exposure is a concern, particularly in younger patients [12].

Phase-resolved functional lung (PREFUL) MRI offers a noninvasive, radiation- and contrast-agent-free imaging modality suited for the detection of subtle lung function alterations across all age groups [13, 14]. A study demonstrated that PREFUL MRI-derived ventilation dynamics distinguished recipients without chronic lung allograft dysfunction from those with early-stage disease, even when regional ventilation and FEV_1_ remained within normal limits [15]. Prior works have additionally shown that PREFUL MRI is capable of monitoring perioperative effects and perfusion alterations in complex clinical conditions [14, 16, 17]. Vogel-Claussen et al. demonstrated that FVL parameters derived from PREFUL MRI were significant predictors of graft loss, whereas conventional PFT measures such as FEV_1_, assessed at the same time point, lacked prognostic value [13]. Collectively, these findings support PREFUL MRI as a promising tool with diagnostic relevance in the context of lung transplant monitoring. However, there remains an unmet clinical need to establish post-transplant baseline values in clinically stable patients, as these have not yet been investigated in comparison with healthy individuals. Establishing such baseline values is a prerequisite for distinguishing physiological variation from disease-related abnormalities and may help to facilitate early detection of regional dysfunction and quantitative perfusion phenotyping after lung transplantation.

The aim of this study was to characterize lung function using PREFUL MRI and spirometry in clinically stable early post-transplant recipients compared with healthy participants.

## Material and methods

### Study design and participants

This prospective, single-center, cross-sectional study was approved by the local ethics committee (No. 9274_BO_S_2020), and written informed consent was obtained from all participants. Between March 2022 and December 2024, bilateral lung transplant recipients were prospectively and consecutively recruited during their inpatient stay following transfer from the intensive care unit. Inclusion criteria were clinically stable graft function, absence of fever, no worsening respiratory symptoms, no hemodynamic instability, no evidence of infection and the ability to lie flat and remain still for the duration of the MRI scan. Participants of all ethnic backgrounds were eligible. Exclusion criteria were single-lung transplantation, contraindications to MRI (e.g., claustrophobia, metallic implants), requirement for continuous oxygen supplementation, status after significant primary graft dysfunction [18], pneumonia, cytomegalovirus reactivation or stroke. Continuous monitoring and comprehensive clinical evaluation were ensured during the entire inpatient stay.

Simultaneously, a control group of healthy participants was recruited and individually matched 1:1 to recipients based on sex and age (± 5 years). Inclusion criteria for the control group included the absence of pulmonary and cardiovascular diseases on clinical examination and anamnesis, normal pulmonary function as confirmed by spirometry, ability to lie flat and remain still for the duration of the MRI scan and no contraindications to MRI. Exclusion criteria were prior smoking, family history of hereditary lung diseases and other known chronic lung diseases [19]. Healthy participants underwent PFT on the same day as their MRI.

Comprehensive clinical data were collected for all transplant recipients, including underlying lung disease, donor characteristics, type of preservation solution used, graft cold ischemic times and the number of intraoperative transfused packed red blood cell units. For a subgroup analysis, lung transplant recipients were prospectively enrolled and randomly assigned to one of two preservation solutions at the time of transplantation (Celsior or Perfadex). Balanced group allocation was ensured prior to surgery with 1:1 sex matching between the two preservation solutions.

### Standardized donor lung preservation protocol

All lungs were perfused under standardized conditions in both antegrade (4 L) and retrograde (1 L) directions. The antegrade perfusion solution was administered over a period of 8–10 min. Iloprost was infused into the pulmonary artery just before perfusion in all included grafts.

Both solutions are classified as extracellular-type preservation fluids but differ substantially in composition and physiological impact. Celsior contains a higher potassium concentration (15 mmol/L) and is enriched with antioxidant molecules such as histidine, mannitol, lactobionate and glutathione. These additives aim to mitigate oxidative stress, limit calcium overload and reduce the development of edema during ischemia and reperfusion. In contrast, Perfadex was specifically developed for pulmonary graft preservation. It is nearly normokalemic (6 mmol/L K^+^) and includes dextran-40, which raises oncotic pressure, decreases erythrocyte aggregation and stabilizes the endothelial–epithelial interface, thereby improving microcirculatory flow [20, 21].

### Pulmonary function testing

Pulmonary function testing was performed in accordance with American Thoracic Society guidelines. Predicted values of forced expiratory volume in one second (FEV_1_), forced vital capacity (FVC) and the FEV_1_-to-FVC ratio were calculated for each participant based on their height, weight, age and sex [22].

### PREFUL MRI

Examinations were conducted on a 1.5-T MRI scanner (MAGNETOM Aera, Siemens Healthcare, Erlangen, Germany) with participants in a head-first supine position. Acquisitions covered the entire lungs and were performed during tidal breathing using a spoiled gradient echo sequence with a high temporal resolution of 192 ms with the following scan parameters for a total of 250 images: repetition time/echo time 3.0/0.82 ms, flip angle 5°, slice thickness 15 mm, slice spacing 15 mm, matrix 128 × 96 interpolated to 264 × 264 and field of view of 500 × 500 mm^2^. Two-fold parallel imaging acceleration using generalized autocalibrating partially parallel acquisitions was applied [23]. The slice package was positioned using the tracheal bifurcation as a reproducible anatomical landmark for the first reference coronal slice. Subsequent slices were systematically positioned in the anterior–posterior direction to achieve complete lung coverage from dorsal to ventral regions. All images underwent a fully automated post-processing pipeline implemented in MATLAB R2022b (MathWorks, Natick, MA) which applied group-oriented image registration for motion compensation and low-pass and high-pass filtering to separate ventilation and perfusion and subsequent phase sorting of registered and filtered images to achieve increased temporal resolution as described previously [19, 24]. Automated segmentation of the pulmonary parenchyma in the reference phase was performed utilizing a U-Net model trained for vessel and lung segmentation [25, 26]. The segmentations were reviewed by a doctoral candidate (M.L.H.) and a radiologist with ten years of expertise in quantitative lung imaging (G.H.P.), both blinded to all clinical data.

### PREFUL MRI-derived parameters

#### Ventilation

Regional ventilation (RVent) was calculated voxel-wise by comparing signal changes for each lung voxel between end-inspiration and end-expiration during tidal breathing [24, 27]. Analogous to PFT, flow-volume loop-correlation metric (FVL) was generated voxel-wise by using data from the entire synthesized breathing cycle for each voxel. Combining the volume of RVent with the dynamic RVent change, loops were compared to a reference loop derived from automatically selected healthy reference region with cross-correlation used as the similarity metric [15].

#### Perfusion

Perfusion was calculated voxel-wise as the difference between the maximum and the minimum signal amplitudes during the synthesized perfusion cycle. By measuring the exchange fraction, the regional blood fraction, the voxel volume and the heart rate, the quantified perfusion was calculated and expressed in milliliters per minute per 100 ml of lung parenchyma [17, 24, 28].

#### Defect percentage maps

Defect percentage maps were generated for the whole lung by calculating the number of defect voxels relative to the total number of lung parenchyma voxels across all coronal sections. Ventilation defect percentages (VDP) maps for RVent were generated by categorizing voxels underneath the 90th percentile multiplied by an empirical factor of 0.4 as RVent defects. Regarding FVL, values below a fixed threshold of 0.9 were regarded as FVL VDP. Perfusion defect percentage (QDP) maps were generated by classifying voxels below the 90th percentile multiplied by a factor of 0.15 as perfusion defects [14, 19, 28].

#### V/Q match and mismatch

Ventilation–perfusion (V/Q) maps were generated for the whole lung voxel-wise by combining binary ventilation data from RVent and FVL with quantified perfusion. V/Q matching was subdivided into two categories: V/Q match healthy representing pulmonary regions with concordant normal ventilation and perfusion and V/Q match defect representing regions where both ventilation and perfusion were impaired. V/Q mismatches highlighted areas with discrepancies between ventilation and perfusion parameters, indicating either exclusive ventilation defects with preserved perfusion or exclusive perfusion defects with preserved ventilation [14, 24].

### Statistical analysis

Sample size estimation was based on a priori power calculation using RVent from previously published data [14]. Assuming a mean difference of 0.0383 and standard deviation of 0.12, a required sample size of 80 subjects (40 per group) was determined [29]. Statistical analyses were performed using JMP Pro18 (SAS Institute, Cary, NC) and MATLAB R2022b. Normality was assessed with the Shapiro–Wilk test. Data are presented in medians and interquartile ranges. Differences between transplant recipients and 1:1 matched healthy controls were assessed using the paired Wilcoxon signed-rank test. For comparisons between preservation solution groups, a separate 1:1 matching among transplant recipients was applied and differences were likewise evaluated using the paired Wilcoxon signed-rank test. The smallest detectable difference for each PREFUL MRI parameter was calculated [29]. Associations between MRI parameters and clinical data were examined using Spearman’s ρ correlation coefficient. A *P* value less than 0.05 represented statistically significant difference for participant demographics and clinical characteristics. A Bonferroni correction was used to adjust for multiple testing across the 11 MRI-derived parameters, resulting in an adjusted significance level of α_adj_ = 0.0045.

## Results

### Participant characteristics

**Fig. 1:**
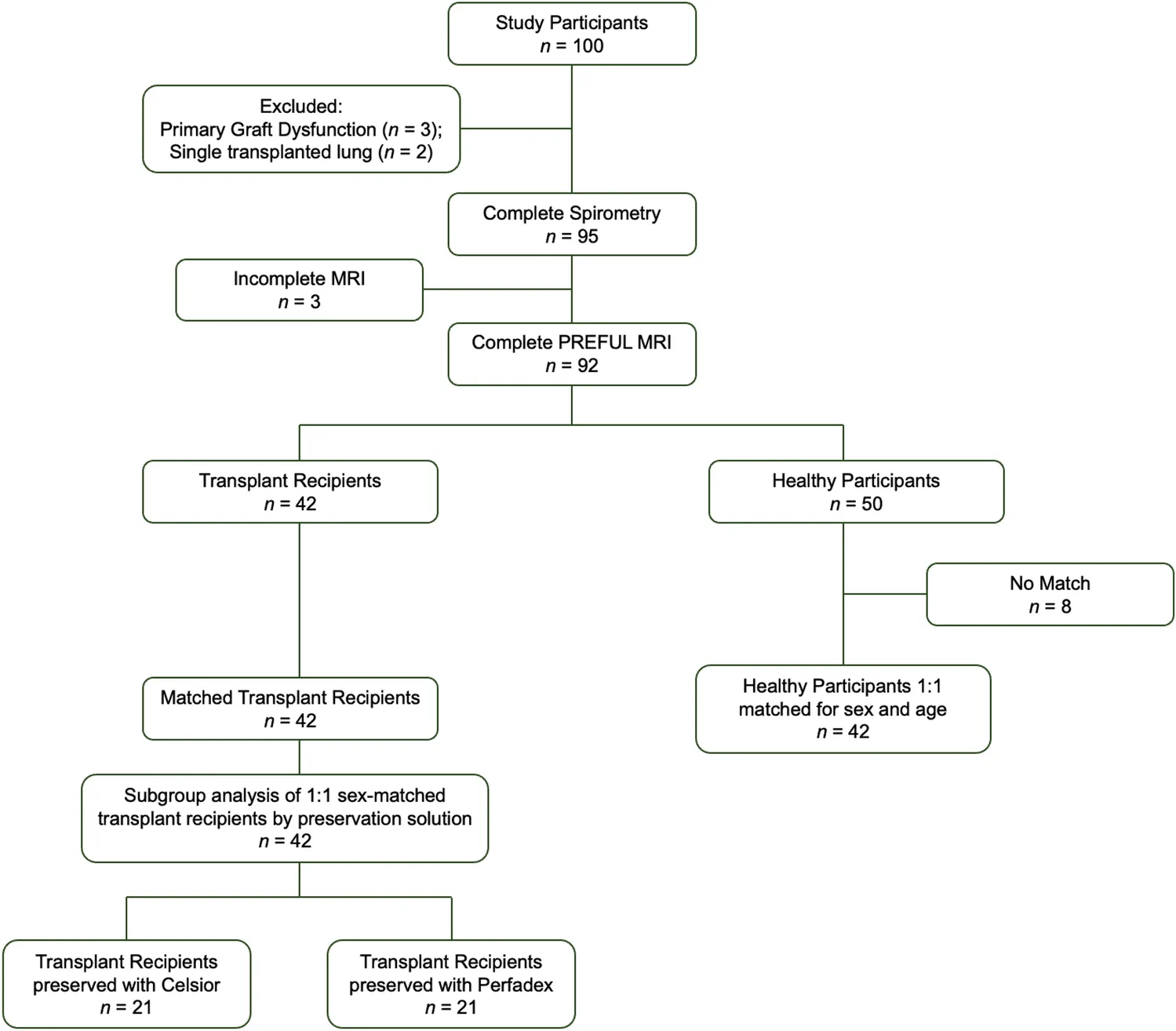
Flowchart summarizes the inclusion process of study participants. PREFUL = phase-resolved functional lung.

Of the 100 initially enrolled participants, five transplant recipients were excluded due to primary graft dysfunction (*n* = 3) and single-lung transplantation (*n* = 2). Three further participants were excluded due to incomplete MRI acquisition caused by claustrophobia, leaving 92 participants with complete PREFUL MRI at a median of 16 days post-transplantation (IQR, 12–20 days). Eight healthy participants could not be matched and were excluded, resulting in a final cohort of 42 transplant recipients (median age 60 years, IQR 54–63; 26 men) and 42 1:1 age- and sex-matched healthy participants (Fig. 1). Demographic characteristics of the entire study population are summarized in Table 1.

**Table 1:** Participant demographics and characteristics

Parameters	Transplant Recipients (*n* = 42)	Healthy Control Participants (*n* = 42)	*P* Value	Transplant Recipients preserved with Celsior (*n* = 21)	Transplant Recipients preserved with Perfadex (*n* = 21)	*P* Value
Sex			0.99^1^			0.99^1^
Male	26 (62%)	26 (62%)		13 (62%)	13 (62%)	
Female	16 (38%)	16 (38%)		8 (38%)	8 (38%)	
Age (y)	60 (54–63)	57 (51–65)	0.16^2^	61 (52–64)	60 (56–63)	0.19^2^
Height (cm)	176 (169–183)	175 (168–182)	0.68^2^	173 (169–181)	176 (167–182)	0.99^2^
Weight (kg)	72 (65–80)	75 (68–88)	**0.003**^2^	70 (65–75)	73 (65–82)	0.88^2^
Body mass index (kg/m^2^)	23 (21–25)	25 (23–28)	**0.002**^**2**^	23 (21–25)	24 (21–26)	0.65^4^
Body surface area (m^2^)	1.9 (1.8–2.0)	1.9 (1.8–2.0)	**0.019**^2^	1.9 (1.8–1.9)	1.9 (1.8–2.0)	0.18^4^
Underlying lung disease						
Obstructive	23 (55%)			10 (48%)	13 (62%)	0.33^2^
Fibrosis	16 (38%)			9 (43%)	7 (33%)	0.49^2^
Other	3 (7.1%)			2 (9.5%)	1 (4.8%)	
Donor characteristics						
Sex						0.76^3^
Male	23 (55%)			11 (52%)	12 (57%)	
Female	19 (45%)			10 (48%)	9 (43%)	
Age (y)	47 (37–58)			47 (38–58)	46 (36–61)	0.85^2^
Height (cm)	175 (170–180)			175 (170–180)	177 (169–180)	0.81^2^
Weight (kg)	80 (70–90)			80 (68–90)	78 (70–87)	0.42^2^
Body mass index (kg/m^2^)	25 (23–29)			26 (23–29)	25 (23–28)	0.48^2^
Body surface area (m^2^)	2.0 (1.8–2.1)			2.0 (1.8–2.1)	1.9 (1.9–2.1)	0.66^2^
Perioperative parameters						
Cold ischemic time of first lung graft (h)	5.60 (4.42–7.00)			6.00 (4.97–7.32)	5.32 (4.45–6.67)	0.32^2^
Cold ischemic time of second lung graft (h)	7.57 (6.38–8.83)			7.90 (6.57–9.53)	6.87 (6.33–8.52)	0.10^2^
Intraoperative transfusion of packed red blood cells (units)	0.0 (0.0–1.0)			0.0 (0.0–1.0)	0.0 (0.0–2.0)	0.78^2^
Post-transplant MRI examination						
Time interval MRI to transplant (d)	16 (12–20)			13 (9–20)	16 (15–20)	0.16^2^
Time interval MRI to PFT (d)	2 (1–5)	0 (0–0)		3 (1–6)	2 (0–4)	0.09^2^
Post-transplant pulmonary function testing						
FEV_1_ (% pred)	71 (53–84)	95 (84–106)	**< 0.001**^2^	70 (51–77)	78 (54–92)	0.13^2^
FVC (% pred)	66 (58–76)	98 (90–108)	**< 0.001**^2^	64 (51–71)	70 (62–81)	0.07^2^
FEV_1_-to-FVC ratio (%)	85 (77–96)	76 (70–81)	**< 0.001**^2^	84 (76–94)	87 (79–98)	0.64^2^

Unless otherwise indicated, data are medians, with IQRs in parentheses, and were compared using the following tests:^1^Fisher’s Exact test and ^2^Wilcoxon signed-rank test. *P* values less than .05 represent statistically significant differences. Body mass index was calculated as body weight in kilograms divided by height in meters squared (kg/m^2^). Body surface area was calculated using the Mosteller formula. Other diagnoses included graft-versus-host disease after bone marrow transplantation for myelodysplastic syndrome, obliterative bronchiolitis and lymphangioleiomyomatosis with tuberous sclerosis. FEV_1_ = forced expiratory volume in 1 s, FVC = forced vital capacity, %pred = percent of the predicted value

### Donor lung preservation

Of the 50 enrolled donor lungs, exclusion criteria were applied as follows: Celsior-preserved lungs were excluded due to primary graft dysfunction (*n* = 2), single-lung transplantation (*n* = 1) and incomplete MRI scans due to claustrophobia (*n* = 1). Perfadex-preserved lungs were excluded due to primary graft dysfunction (*n* = 1), single-lung transplantation (*n* = 1) and incomplete MRI scans due to claustrophobia (*n* = 2). This resulted in 21 lungs preserved with Celsior and 21 with Perfadex for analysis. No significant differences in demographics or spirometry were observed between preservation solution groups.

### Pulmonary function testing

Transplant recipients underwent pulmonary function testing and MRI with a median interval of 2 days (IQR, 1–5), whereas healthy participants completed spirometry on the same day as their MRI. Predicted FEV_1_, predicted FVC and FEV_1_-to-FVC ratio were all reduced in transplant recipients compared with healthy participants (all *P* < 0.001).

### PREFUL MRI parameters in lung transplant recipients and matched healthy control participants

**Fig. 2:**
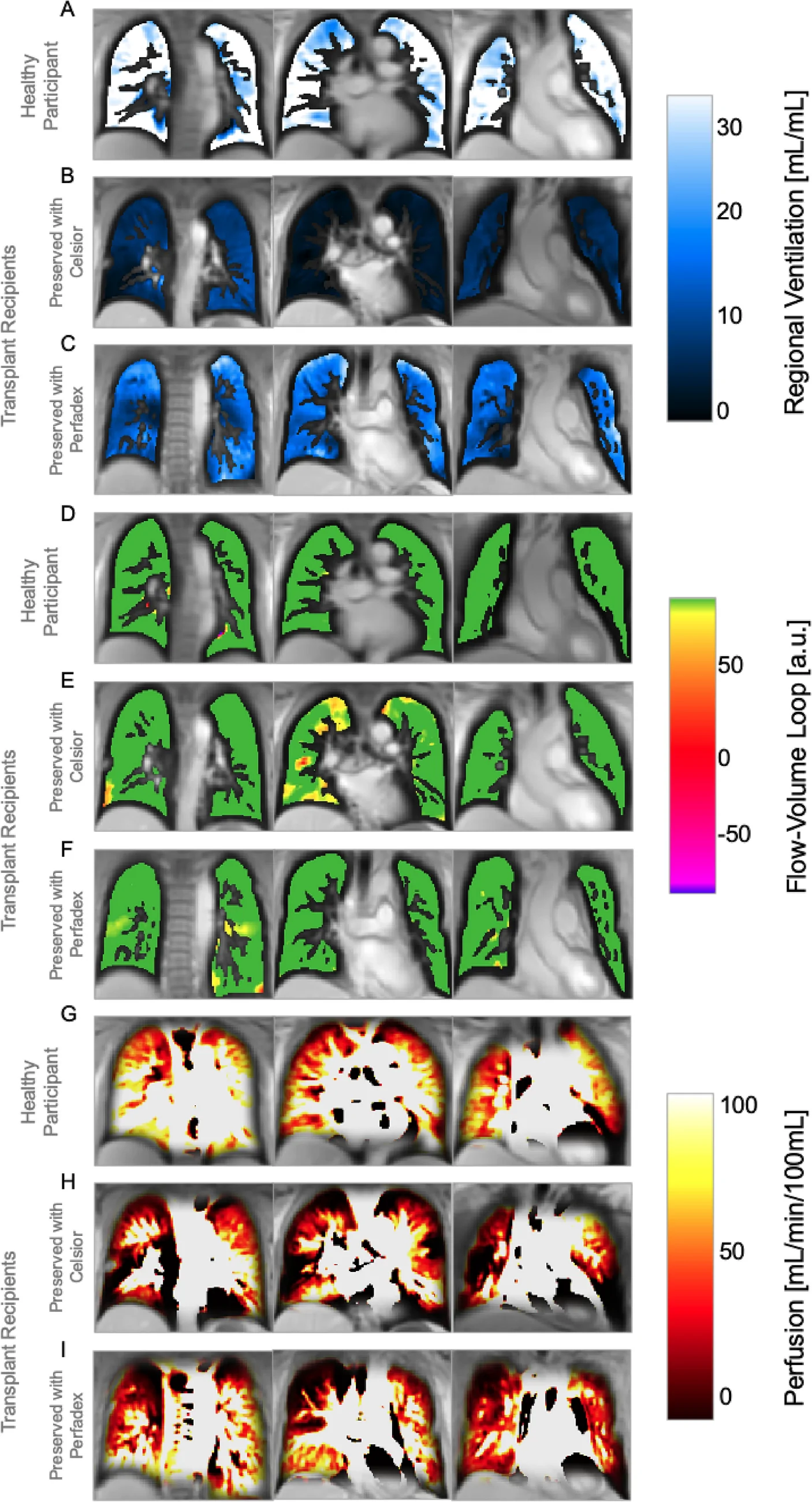
Images show exemplary coronal sections of phase-resolved functional lung MRI ventilation and perfusion maps in an age-(± 5 years) and sex-matched healthy participant and the corresponding 64-year-old female lung transplant recipient preserved with Celsior and a 61-year-old female transplant recipient preserved with Perfadex. **A–C** Images show regional ventilation, with values of **A** 30% in the healthy control participant, **B** 4% in the Celsior-preserved transplant participant and **C** 13% in the transplant recipient preserved with Perfadex. **D–F** Images show the flow-volume loop-correlation metric, with **D** the healthy control participant at 98%, **E** the transplant participant with Celsior at 94% and **F** 96% in the Perfadex-preserved transplant recipient. **G–I** Images show perfusion maps depicting a median perfusion of 68 mL/min per 100 mL for the healthy control participant, 35 mL/min per 100 mL for the transplant recipient with Celsior and 41 mL/min per 100 mL for the Perfadex-preserved participant

**Fig. 3:**
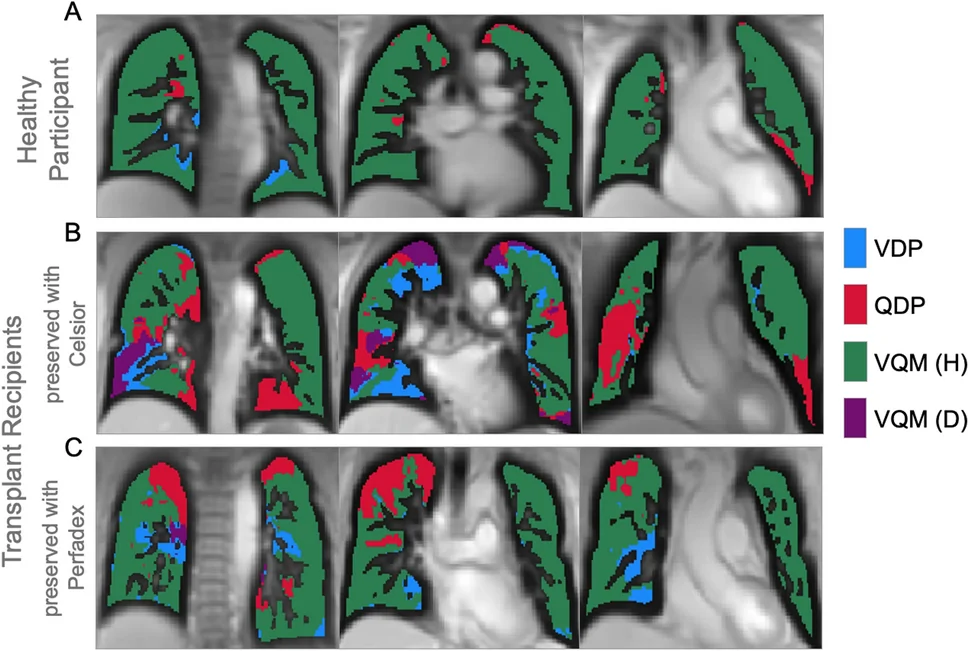
Images show exemplary coronal sections of phase-resolved functional lung MRI defect maps in an age-(± 5 years) and sex-matched healthy participant, the corresponding 64-year-old female lung transplant recipient preserved with Celsior and a 61-year-old female transplant recipient preserved with Perfadex. **A** Images in a healthy control participant, with a ventilation defect percentage (VDP) of 2%, a perfusion defect percentage (QDP) of 3%, a ventilation/perfusion (V/Q) match healthy percentage (VQM [H]) of 95% and no V/Q mismatch defect (VQM [D]). **B** Images in a Celsior-preserved lung transplant recipient show a ventilation defect percentage of 9%, a perfusion defect percentage of 25%, a VQM [H] of 69% and VQM [D] of 3%. **C** Images in a Perfadex-preserved transplant recipient show VDP of 7%, QDP of 15%, VQM [H] of 79% and VQM [D] of 1%

**Fig. 4:**
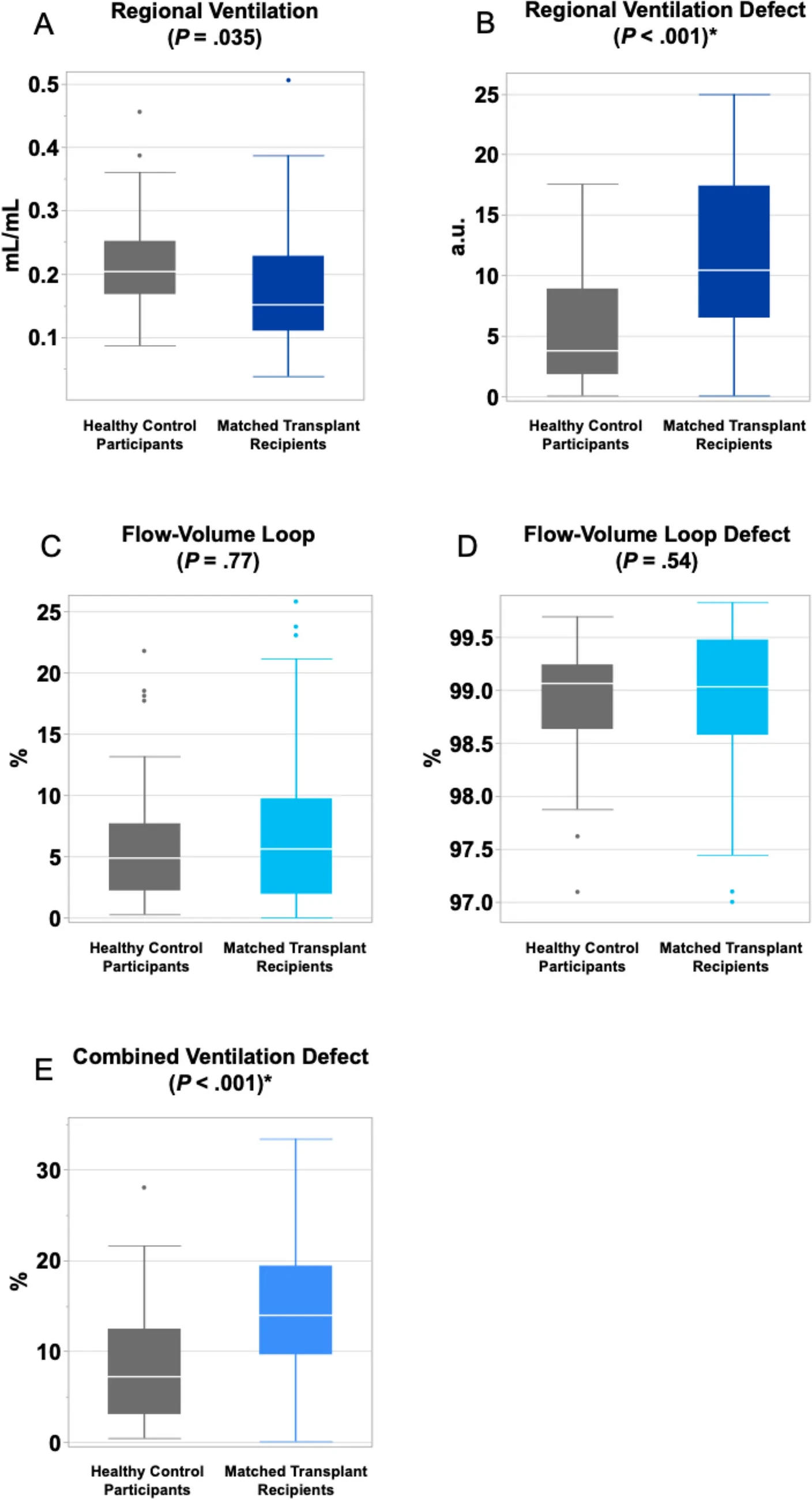
Comparisons of phase-resolved functional lung MRI-derived ventilation parameters, ventilation–perfusion (V/Q) matches and V/Q mismatches between age- (± 5 years) and sex-matched healthy participants and lung transplant recipients at a median of 16 days (IQR, 12–20) post-transplantation. **A+B** Box plots show regional ventilation parameters. **C+D** Box plots show flow-volume loops (FVL). **E** Box plot shows the combined defect map of regional ventilation and FVL. * = Statistically significant differences after Bonferroni correction for 11 comparisons (*P* < 0.0045). The line inside each box indicates the median, and the box represents the IQR from the 25th to the 75th percentile. Whiskers mark the smallest and largest values within 1.5 times the IQR, and points beyond this range are considered outliers. au = arbitrary units

**Fig. 5:**
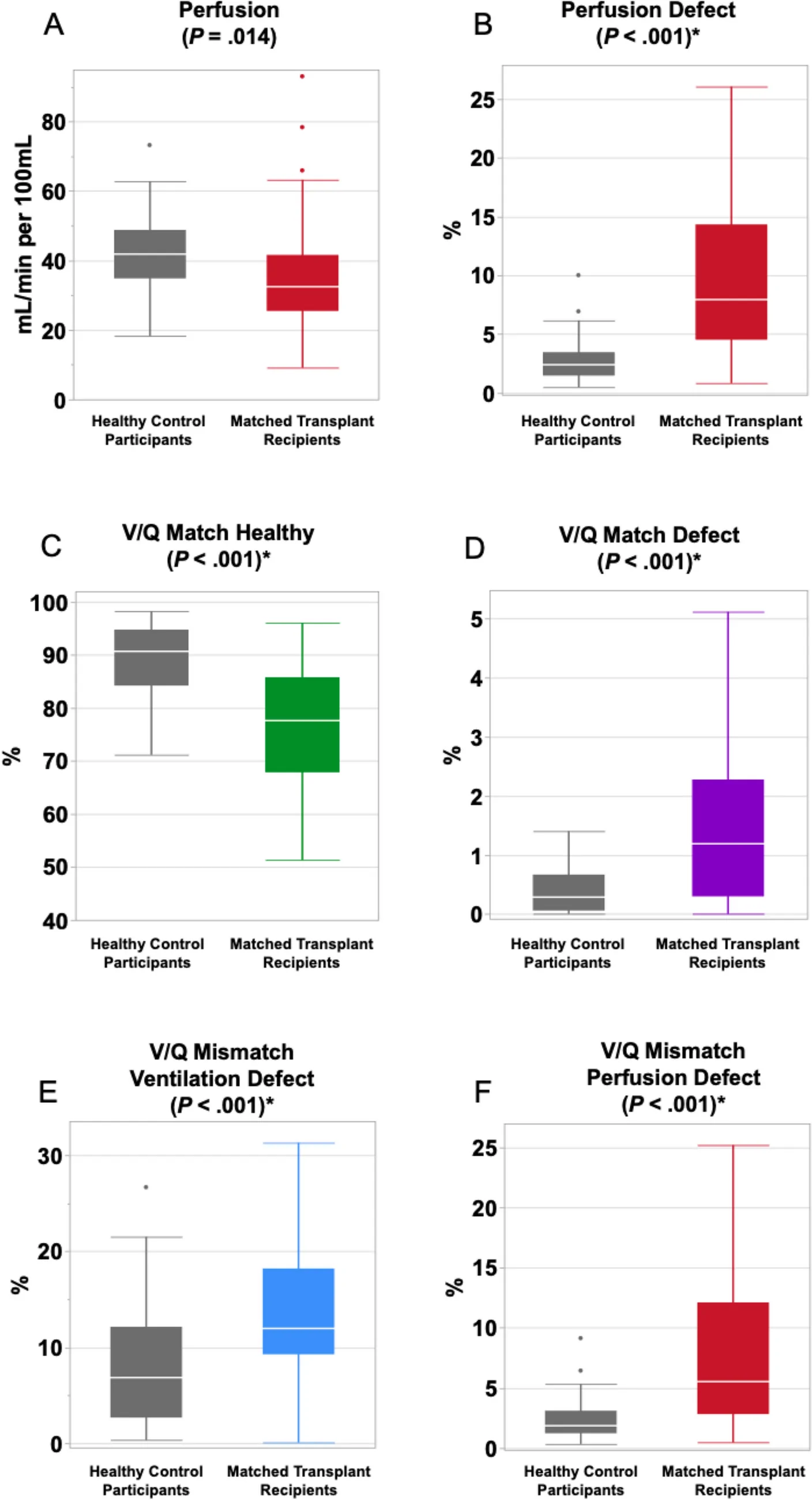
Comparisons of phase-resolved functional lung MRI-derived perfusion parameters, ventilation–perfusion (V/Q) matches and V/Q mismatches between age- (± 5 years) and sex-matched healthy participants and lung transplant recipients at a median of 16 days (IQR, 12–20) post-transplantation. **A+B** Box plots show perfusion parameters and **C–F** box plots show V/Q matches and mismatches. * = Statistically significant differences after Bonferroni correction for 11 comparisons (*P* < 0.0045). The line inside each box indicates the median, and the box represents the IQR from the 25th to the 75th percentile. Whiskers mark the smallest and largest values within 1.5 times the IQR, and points beyond this range are considered outliers

A median of 5 coronal MRI slices per participant was acquired (IQR, 3–6). A comparison of PREFUL MRI parameters and healthy controls is shown in Table 2 (Figs. 2 and 3). Transplant recipients showed an elevated RVent VDP, VDP_combined_ and QDP, all with *P* < 0.001 (Figs. 4, 5). V/Q matching parameters revealed lower healthy V/Q matching in transplant recipients than in healthy controls (*P* < 0.001). V/Q match of defects was higher in transplant recipients (*P* < 0.001) QDP was higher in the Celsior subgroup compared with the Perfadex subgroup (*P* = 0.004), while healthy V/Q matching was reduced in the Celsior group (*P* < 0.001).

**Table 2:** Comparison of PREFUL parameters among bilateral lung transplant recipients preserved with celsior or perfadex and healthy participants

MRI Parameters	Transplant Recipients (*n* = 42)	1:1 Matched Healthy Participants (*n* = 42)	*P* Value	Statistically smallest detectable difference for *n* = 42	Transplant Recipients preserved with Celsior (*n* = 21)	Transplant Recipients preserved with Perfadex (*n* = 21)	*P* Value	Statistically smallest detectable difference for *n* = 21
	Median (IQR)	Median (IQR)			Median (IQR)	Median (IQR)		
Ventilation								
RVent (mL/mL)	0.15 (0.11–0.23)	0.20 (0.17–0.25)	0.03	0.05	0.15 (0.11–0.25)	0.15 (0.11–0.20)	0.68	0.08
RVent VDP (%)	10 (6.5–17)	3.7 (1.8–8.9)	**< 0.001**	3.6	14 (9.0–19)	8.3 (4.2–13)	0.02	5.3
FVL (au)	99.0 (98.6–99.5)	99.1 (98.6–99.2)	0.77	0.36	99.1 (97.9–99.4)	98.9 (98.6–99.5)	0.68	0.53
FVL VDP (%)	5.6 (2.0–9.8)	4.8 (2.3–7.7)	0.54	3.3	6.0 (2.3–15)	5.1 (1.6–8.4)	0.25	4.8
VDP_combined_ (RVent, FVL) (%)	14 (9.7–19.4)	7.1 (3.0–13)	**< 0.001**	4.1	19 (12–24)	12 (6.9–15)	0.01	6.0
Perfusion								
Perfusion (mL/min/100 mL)	33 (26–41)	42 (35–49)	0.01	8.9	32 (25–40)	33 (25–43)	0.50	13
QDP (%)	7.9 (4.5–14)	2.4 (1.5–3.4)	**< 0.001**	3.2	13 (7.7–20)	5.5 (3.1–8.3)	**0.004**	4.6
V/Q ratio								
V/Q match healthy (%)	78 (68–86)	91 (84–95)	**< 0.001**	4.9	69 (65–77)	85 (78–89)	**< 0.001**	7.1
V/Q match defect (%)	1.2 (0.30–2.3)	0.29 (0.06–0.67)	**< 0.001**	0.62	1.6 (0.69–2.9)	0.65 (0.13–1.4)	0.02	0.90
V/Q mismatch (exclusive ventilation defect) (%)	12 (9.2–18)	6.8 (2.7–12)	**< 0.001**	3.7	16 (11–20)	11 (6.2–13)	0.02	5.4
V/Q mismatch (exclusive perfusion defect) (%)	5.6 (2.8–12)	1.9 (1.2–3.1)	**< 0.001**	3.0	12 (4.8–17)	4.1 (2.5–6.4)	0.01	4.4

Unless otherwise indicated, data are medians; data in parentheses are IQRs. Combined parameters were calculated as means. Regional ventilation and flow-volume loop-correlation metric defect maps were combined by using a logical formula or operation to calculate combined ventilation volumes. Bonferroni correction for multiple comparisons (*n* = 11 MRI parameters) was applied to the *P* values: *P* < 0.0045 indicates statistically significant differences; *P* values were calculated using Wilcoxon signed-rank test. Data are from coronal sections. au = arbitrary units, FVL = flow-volume loop, PREFUL = phase-resolved functional lung, QDP = perfusion defect percentage, RVent = regional ventilation, VDP = ventilation defect percentage, V/Q = ventilation-perfusion

### Correlation of PREFUL MRI parameters with pulmonary function testing

Correlation was assessed between PREFUL MRI parameters and PFT in Table 3. FVL had a positive correlation with FEV_1_-to-FVC ratio (*ρ* = 0.47, *P* = 0.002) and FVL VDP correlated inversely with the FEV_1_-to-FVC ratio (*ρ* = − 0.44, *P* = 0.004). QDP was inversely correlated with FEV_1_ (*ρ* = − 0.49, *P* = 0.001). V/Q match defect showed an inverse correlation with FEV_1_ (*ρ* = –0.48, *P* = 0.003) and with the FEV_1_-to-FVC ratio (*ρ* = − 0.58, *P* < 0.001).

**Table 3:** Correlation between phase-resolved functional lung MRI parameters and pulmonary function testing in early lung transplant recipients

Parameters	FEV_1_ (%pred)		FVC (%pred)		FEV_1_-to-FVC ratio (%)	
	*ρ*	*P* Value	*ρ*	*P* Value	*ρ*	*P* Value
**Transplant Recipients** (*n* = 42)						
*Ventilation*						
RVent (mL/mL)	0.08	0.60	− 0.01	0.97	0.35	0.02
RVent VDP (%)	− 0.12	0.44	− 0.08	0.61	− 0.18	0.27
FVL (au)	0.20	0.20	− 0.05	0.77	**0.47**	**0.002**
FVL VDP (%)	− 0.22	0.17	− 0.03	0.86	**− 0.44**	**0.004**
VDP_combined_ (RVent, FVL) (%)	− 0.24	0.12	− 0.12	0.44	− 0.39	0.01
*Perfusion*						
Perfusion (mL/min per 100 mL)	− 0.01	0.96	− 0.02	0.88	− 0.09	0.58
QDP (%)	**− 0.49**	**0.001**	− 0.38	0.01	− 0.29	0.07
*V/Q ratio*						
V/Q match healthy (%)	0.38	0.01	0.25	0.11	0.39	0.01
V/Q match defect (%)	**− 0.48**	**0.003**	− 0.26	0.10	**− 0.58**	**< 0.001**
V/Q mismatch (exclusive ventilation defect) (%)	− 0.20	0.21	− 0.07	0.65	− 0.34	0.03
V/Q mismatch (exclusive perfusion defect) (%)	− 0.42	0.01	− 0.32	0.04	− 0.20	0.21
**Healthy Control Participants** (*n* = 42)						
*Ventilation*						
RVent (mL/mL)	0.13	0.40	0.26	0.10	0.20	0.21
RVent VDP (%)	− 0.15	0.35	− 0.16	0.31	− 0.18	0.26
FVL (au)	0.14	0.36	0.05	0.76	0.19	0.22
FVL VDP (%)	− 0.38	0.01	− 0.35	0.03	− 0.14	0.38
VDP_combined_ (RVent, FVL) (%)	− 0.24	0.12	− 0.30	0.05	− 0.11	0.47
*Perfusion*						
Perfusion (mL/min/100 mL)	− 0.12	0.45	− 0.18	0.25	0.17	0.29
QDP (%)	0.01	0.98	− 0.18	0.26	0.10	0.52
*V/Q ratio*						
V/Q match healthy (%)	0.25	0.11	0.33	0.04	0.09	0.59
V/Q match defect (%)	− 0.23	0.14	− 0.27	0.09	− 0.16	0.31
V/Q mismatch (exclusive ventilation defect) (%)	− 0.24	0.12	− 0.29	0.06	− 0.12	0.47
V/Q mismatch (exclusive perfusion defect) (%)	0.11	0.47	− 0.07	0.67	0.18	0.26

Regional ventilation and flow-volume loop-correlation metric defect maps were combined by using a logical formula or operation to calculate combined ventilation volumes. *P* values were calculated using Spearman’s ρ and Bonferroni correction for multiple comparisons (*n* = 11 MRI parameters) was applied to the *P* values: *P* < 0.0045 indicates statistically significant differences; data are from coronal sections. au = arbitrary units, FEV_1_ = forced expiratory volume in 1 s, FVC = forced vital capacity, %pred = percent of the predicted value, FVL = flow-volume loop, PREFUL = phase-resolved functional lung, QDP = perfusion defect percentage, RVent = regional ventilation, VDP = ventilation defect percentage, V/Q = ventilation-perfusion

### Correlation of PREFUL MRI parameters and pulmonary function testing with donor characteristics and perioperative parameters

Cold ischemic time of the first lung graft was associated with QDP (*ρ* = 0.43, *P* = 0.004) and with V/Q mismatch (exclusive perfusion defect) (*ρ* = 0.48, *P* = 0.001), Table 4. Similarly, the cold ischemic time of the second graft correlated with V/Q mismatch (exclusive perfusion defect) (*ρ* = 0.46, *P* = 0.002).

**Table 4:** Correlation between phase-resolved functional lung MRI parameters, pulmonary function testing and cold ischemic time of the first and second lung graft in lung transplant recipients

MRI parameters	Donor Characteristics and Perioperative Parameters									
	Donor Age		Donor BMI		Cold Ischemic Time of the First Lung Graft		Cold Ischemic Time of the Second Lung Graft		Intraoperative Transfusion of Packed Red Blood Cells	
	*ρ*	*P* Value	*ρ*	*P* Value	*ρ*	*P* Value	*ρ*	*P* Value	*ρ*	*P* Value
Ventilation										
RVent (mL/mL)	0.08	0.64	− 0.29	0.06	− 0.07	0.66	− 0.12	0.44	0.05	0.74
RVent VDP (%)	0.21	0.19	− 0.01	0.97	0.15	0.35	0.17	0.29	− 0.04	0.79
FVL (au)	− 0.35	0.03	− 0.19	0.24	0.15	0.35	0.12	0.43	0.05	0.74
FVL VDP (%)	0.27	0.08	0.10	0.53	0.02	0.90	0.03	0.85	− 0.01	0.94
VDP_combined_ (RV, FVL) (%)	0.25	0.10	0.03	0.84	0.12	0.46	0.14	0.37	− 0.10	0.54
Perfusion										
Perfusion (mL/min/100 mL)	0.01	0.96	0.10	0.53	− 0.04	0.79	− 0.06	0.70	− 0.28	0.08
QDP (%)	0.05	0.74	0.10	0.55	**0.43**	**0.004**	0.41	0.01	− 0.36	0.02
V/Q ratio										
V/Q match healthy (%)	− 0.21	0.18	− 0.13	0.41	− 0.30	0.05	− 0.31	0.05	0.32	0.04
V/Q match defect (%)	0.18	0.27	0.08	0.64	0.17	0.28	0.15	0.35	− 0.24	0.13
V/Q mismatch (exclusive ventilation defect) (%)	0.25	0.11	0.04	0.79	0.12	0.45	0.16	0.32	− 0.05	0.76
V/Q mismatch (exclusive perfusion defect) (%)	0.02	0.89	0.07	0.66	**0.48**	**0.001**	**0.46**	**0.002**	− 0.29	0.07
PFT parameters										
FEV_1_ (% pred)	− 0.16	0.30	− 0.32	0.04	− 0.09	0.56	− 0.07	0.68	− 0.03	0.86
FVC (% pred)	− 0.10	0.55	− 0.29	0.06	− 0.08	0.63	− 0.07	0.66	0.02	0.92
FEV_1_-to-FVC ratio (%)	− 0.24	0.12	− 0.01	0.97	− 0.04	0.81	0.02	0.88	− 0.04	0.79

Regional ventilation and flow-volume loop-correlation metric defect maps were combined by using a logical formula or operation to calculate combined ventilation volumes. Bonferroni correction for multiple comparisons was applied to the *P* values: *P* < 0.0045 indicates statistically significant differences; *P* values were calculated using Spearman’s ρ. Data are from coronal sections. Body mass index (BMI) was calculated as body weight in kilograms divided by height in meters squared, au = arbitrary units, FVL = flow-volume loop, PFT = pulmonary function testing, PREFUL = phase-resolved functional lung, QDP = perfusion defect percentage, RVent = regional ventilation, VDP = ventilation defect percentage, V/Q = ventilation-perfusion, FEV_1_ = forced expiratory volume in 1 s, FVC = forced vital capacity, %pred = percent of the predicted value

## Discussion

In this prospective study, we investigated early pulmonary function in clinically stable lung transplant recipients using phase-resolved functional lung MRI parameters and spirometry. Transplant recipients were compared with 1:1 age- and sex-matched healthy participants. Our results showed elevated defect percentages as well as pronounced ventilation–perfusion mismatch in transplant recipients compared with controls, with defect percentages correlating with spirometry and perfusion demonstrating a correlation with cold ischemic time. To our knowledge, these findings represent the first systematic evaluation of functional lung baseline values in a comprehensive, thoroughly defined sample of lung transplant recipients without complications and healthy control participants using an advanced MRI approach.

PFT constitutes the established clinical standard for post-transplant graft surveillance owing to its widespread availability and well-validated diagnostic value for global lung function assessment [30, 31]. In our cohort, predicted FEV_1_ and FVC were reduced in transplant recipients compared with healthy controls, confirming the expected global ventilatory changes in the early postoperative period and supporting the continued value of spirometry as the clinical reference standard. PREFUL MRI extends this functional characterization by resolving regional ventilation and perfusion at the voxel level, providing access to regional V/Q information that is, by design, beyond the spatial scope of any global flow-volume measurement [16, 19, 24]. While whole lung regional ventilation, flow-volume loop and perfusion parameters did not differ between recipients and healthy controls, recipients exhibited markedly elevated defect maps. VDP_combined_ was approximately twice as high in transplant recipients as in healthy controls, potentially reflecting underlying structural abnormalities, including mild or partial atelectasis as part of the post-surgical recovery process [32]. Transplant recipients also showed perfusion defects that were more than threefold elevated. To our knowledge, this pattern of inhomogeneous pulmonary microcirculation has not previously been reported in vivo. One explanation might be that the pulmonary microcirculation of the allograft is uniquely vulnerable during the early post-transplant period. There is experimental and clinical evidence that endothelial damage, elevated pulmonary vascular resistance and impaired recovery of microvascular tone are early consequences of ischemia–reperfusion injury, each of which may, at this stage, reduce regional perfusion in otherwise well-ventilated lung units [33–35]. An additional contributing factor may be ongoing right ventricular impairment, which can further modulate perfusion distribution patterns [24].

Regional alterations were also evident in V/Q ratios, where V/Q matches of healthy lung parenchyma, defects and V/Q mismatch (exclusive ventilation, perfusion) differed from those observed in healthy participants. Transplant recipients exhibited lower healthy V/Q matching and more than fourfold higher rates of concordant V/Q match defects, reflecting simultaneous co-localized impairment of both ventilation and perfusion, most plausibly representing parenchymal consolidation, atelectasis or areas of ischemia–reperfusion-induced alveolar–capillary disruption [9, 36]. Both isolated V/Q mismatch patterns were substantially more prevalent in transplant recipients. Collectively, the ventilation and perfusion defect maps and V/Q ratios observed in clinically stable transplant recipients can be quantitatively positioned between values reported in healthy individuals and those observed in patients with established chronic lung disease [13, 16, 37]. This functional V/Q profiling may constitute a fingerprint of the early post-transplant allograft that extends the information provided by spirometry toward regional parenchymal resolution.

In the lung transplant recipient cohort, several PREFUL MRI parameters demonstrated significant correlations with spirometric lung function, including FVL, FVL VDP, QDP and V/Q ratios, all correlating with FEV_1_-to-FVC ratio or FEV_1_. The absence of significant correlations for RVent-based parameters, while FVL-based metrics reached significance, is in line with prior PREFUL studies. In COPD, FVL-derived parameters have been shown to correlate more strongly with FEV_1_ than static RVent, as tidal flow-volume loop analysis captures dynamic airway behavior more analogous to spirometric maneuvers [38]. Similarly, in severe asthma, FVL VDP yielded a stronger correlation with FEV_1_ than RVent VDP [39, 40]. No significant correlations of defect percentages were observed in the healthy control group. This may partly be explained by the low VDP values in healthy volunteers (approximately 3.7% for RVent and 4.8% for FVL), leaving insufficient dynamic range in either variable to yield detectable associations.

Reduced perfusion and exclusive perfusion defects as a component of V/Q mismatch were associated with prolonged cold ischemic time of the implanted lung allografts. Impairment confined to the perfusion compartment without a corresponding ventilatory deficit suggests a primary vascular mechanism, which may be partially explained by ischemia–reperfusion injury. In this context, ischemia–reperfusion preferentially targets the pulmonary microvascular endothelium through oxidative burst, neutrophil adhesion and endothelial barrier disruption, culminating in microvascular obstruction and impaired capillary recruitment [33–35]. Prolonged cold ischemic time is an established independent predictor of acute allograft rejection [41], a relationship corroborated in a propensity score-matched national cohort of 6,422 recipients where it independently predicted both acute rejection and reduced long-term survival [6]. Conventional spirometric parameters were not significantly associated with cold ischemic time in our cohort, suggesting that PREFUL-derived perfusion defect maps may serve as a complementary noninvasive correlate of ischemic allograft burden not accessible through global flow-volume analysis alone. However, their value as predictive imaging biomarkers of acute allograft rejection and reduced long-term survival remains to be investigated in future studies.

In a 1:1 sex-matched subgroup analysis, recipients of Celsior-preserved lungs exhibited higher QDP and lower healthy V/Q matching compared with Perfadex-preserved lung recipients. To the best of our knowledge, this imaging-based observation has not been previously reported. Since Perfadex contains dextran-40, which maintains oncotic pressure, reduces erythrocyte aggregation and exerts antithrombotic effects within the pulmonary microcirculation, it potentially contributes to the preservation of endothelial integrity during ischemia [20, 21, 42]. As a further explanation, Celsior’s higher potassium concentration and distinct antioxidant profile may attenuate calcium overload and oxidative stress during reperfusion via complementary cellular mechanisms [20, 42]. These preliminary imaging-based findings from a subgroup analysis warrant further investigation in prospective, multicenter trials. Furthermore, a comparison with photon-counting computed tomography-derived imaging parameters might enable a complementary, multimodal assessment of regional lung function in the early post-transplant period [43]. Of particular clinical relevance, allograft rejection frequently manifests with spatially heterogeneous parenchymal involvement [44], which inherently limits the sensitivity of conventional diagnostic approaches. Current transbronchial sampling is typically performed at anatomically predefined sites, most commonly the right lower lobe [11, 45]. Beyond quantitative surveillance, functional lung MRI-derived imaging maps may enable a future clinical pathway toward image-informed transbronchial biopsy [8, 41]. Specifically, PREFUL MRI-derived VDP and QDP maps could facilitate targeted sampling of functionally compromised regions, potentially improving diagnostic yield in selected cases.

Our study has several limitations. The single-center design and strict inclusion of clinically stable recipients limit generalizability to the broader and more heterogeneous transplant population. The cross-sectional acquisition precludes characterization of the longitudinal functional recovery trajectory and limits causal inference regarding the directionality of observed associations. The subgroup analysis comparing preservation solutions was exploratory and residual confounding cannot be excluded given the sample size. Differences in body composition between recipients and healthy controls may have introduced residual confounding not fully adjustable within this cohort.

In conclusion, phase-resolved functional lung MRI detected regional ventilation and perfusion heterogeneities in clinically stable lung transplant recipients early after transplantation and MRI-based pulmonary dysfunction correlated with spirometry and cold ischemic time. These findings may improve our understanding of physiologic effects of lung recovery post-transplant and lay a foundation for future research of predictive imaging biomarkers in this complex patient population. Further investigations should evaluate the course of lung alterations at various stages after lung transplantation in patients with complications such as acute rejection, potentially coordinated with transbronchial biopsy intervals.

## Abbreviations

FEV_1_: Forced expiratory volume in the first second of expiration
FVC: Forced vital capacity
FVL: Flow-volume loop
PFT: Pulmonary function testing
PREFUL: Phase-resolved functional lung
QDP: Perfusion defect percentage
RVent: Regional ventilation
VDP: Ventilation defect percentage
V/Q: Ventilation/perfusion
